# Psychiatric diagnoses in older people with intellectual disability in comparison with the general population: a register study

**DOI:** 10.1017/S2045796017000051

**Published:** 2017-02-23

**Authors:** A. Axmon, P. Björne, L. Nylander, G. Ahlström

**Affiliations:** 1 Department of Occupational and Environmental Medicine, Lund University, SE-221 00 Lund, Sweden; 2 Research and Development Unit, City Office, City of Malmö, Sweden; 3 Department of Clinical Sciences/Psychiatry, Lund University, Lund, Sweden; 4 Gillberg Neuropsychiatry Centre, University of Gothenburg, Göteborg, Sweden; 5 Department of Health Sciences, Lund University, Lund, Sweden

**Keywords:** Diagnosis, elderly, mental health, mental retardation

## Abstract

**Aims.:**

To describe the occurrence of psychiatric diagnoses in a specialist care setting in older people with intellectual disability (ID) in relation to those found in the same age group in the general population.

**Method.:**

A cohort of people with ID (*n* = 7936), aged 55 years or more in 2012, was identified, as was an age and sex-matched cohort from the general population (*n* = 7936). Information regarding psychiatric diagnoses during 2002–2012 was collected from the National Patient Register, which contains records from all inpatient care episodes and outpatient specialist visits in Sweden. The mean age at the start of data collection (i.e. January 1st, 2002) was 53 years (range 44–85 years).

**Results.:**

Seventeen per cent (*n* = 1382) of the people in the ID cohort had at least one psychiatric diagnosis recorded during the study period. The corresponding number in the general population cohort was 10% (*n* = 817), which translates to an odds ratio (OR) of 1.84. The diagnoses recorded for the largest number of people in the ID cohort were ‘other’ (i.e. not included in any of the diagnostic groups) psychiatric diagnoses (10% of the cohort had at least one such diagnosis recorded) and affective disorders (7%). In the general population cohort, the most common diagnoses were affective disorders (4%) and alcohol/substance-abuse-related disorders (4%). An increased odds of having at least one diagnosis was found for all investigated diagnoses except for alcohol/substance-abuse-related disorders (OR = 0.56). The highest odds for the ID cohort was found for diagnosis of psychotic disorder (OR = 10.4) followed by attention deficit/hyperactive disorder (OR = 3.81), dementia (OR = 2.71), personality disorder (OR = 2.67), affective disorder (OR = 1.74) and anxiety disorder (OR = 1.36). People with ID also had an increased odds of psychiatric diagnoses not included in any of these groups (OR = 8.02). The percentage of people with ID who had at least one diagnosis recorded during the study period decreased from more than 30% among those aged 55–59 years in 2012 (i.e. born 1953–1957) to approximately 20% among those aged 75+ years in 2012 (i.e. born in or before 1937).

**Conclusions.:**

Older people with ID seem to be more likely to have psychiatric diagnoses in inpatient or outpatient specialist care than their peers in the general population. If this is an effect of different disorder prevalence, diagnostic difficulties or differences in health care availability remains unknown. More research is needed to understand the diagnostic and treatment challenges of psychiatric disorders in this vulnerable group.

## Introduction

The number of people with intellectual disability (ID) who reach older age is increasing (Haveman, 2004; Coppus, 2013; Dieckmann *et al.* 2015). Moreover, the ageing process starts at a younger age among people with ID (WHO, 2000), and they show signs of frailty earlier than the general population (Evenhuis *et al.* 2012). A few studies that have focused on psychiatric diagnoses among older people with ID suggest that old age increases the risk of overall psychiatric morbidity, dementia, anxiety disorder and depression (Cooper, 1997*a*, *b*; Deb *et al.* 2001). In wider and/or younger age groups, people with ID may have a higher risk for psychiatric disorders than the general population (Bhaumik *et al.* 2008; Nettelbladt *et al.* 2009; Yoo *et al.* 2012). If this can be extrapolated into older age groups is uncertain.

An accurate psychiatric diagnosis for a person with ID can be difficult to make, due to e.g. communication difficulties, physical health issues (Bhaumik *et al.* 2008), diagnostic overshadowing (Reiss & Szyszko, 1983) and lack of assessment tools adapted for people with ID (Alexander & Cooray, 2003; Moreland *et al.* 2008; Hermans & Evenhuis, 2010). This is most likely true for all psychiatric diagnoses for which there are no objective markers, i.e. with disorders due to alcohol or substance use as the only exception. Due to this, a large part of psychiatric morbidity in people with ID may be hidden (Salvador-Carulla *et al.* 2000), and it has been suggested that people with ID do not receive the services that their health conditions require (Ouellette-Kuntz *et al.* 2005).

Having one psychiatric disorder seems to be a risk factor for more psychiatric disorders among people with ID (Goldberg *et al.* 1995; Lidher *et al.* 2005; Bakken *et al.* 2010). Some psychiatric disorders have also been suggested to be associated with higher risk of physical disorders (e.g. Moss & Patel, 1997; Cooper, 1999). Further, psychiatric disorders such as schizophrenia-spectrum psychoses appear to be more debilitating among people with ID (Bouras *et al.* 2004). Thus, failure to correctly diagnose psychiatric disorders in people with ID may have severe consequences for the individual.

By assessing patterns of psychiatric diagnoses in older people with ID, and comparing these to those in the general population, important knowledge can be made available to policy makers, health organizations and service providers. This knowledge may then provide the basis for improvements in support and service for ageing people with ID.

The aim of the present study was to describe the occurrence of psychiatric diagnoses in a specialist care setting in older people with ID in relation to those found in the same age group in the general population.

## Material and methods

This was a register-based study investigating psychiatric diagnoses among older people with ID in comparison with same-aged people from the general population. The study cohorts were defined using national registers, and outcome data were collected from national registers.

### Study population

In Sweden, people with an ID or autism spectrum disorder (ASD) can apply for services according to the Act Concerning Support and Service for People with Certain Functional Impairments (Swedish abbreviation: LSS) (SFS 1993:387, 1993). People with a diagnosis of ID or ASD are eligible to apply for support through a case manager in the municipality. All support provided by the municipality is documented in the *LSS-register*, which is managed by the Swedish National Board of Health and Welfare, a government agency under the Ministry of Health and Social Affairs.

In the present study, we used LSS support as a proxy for ID. Through the LSS-register, all people with at least one form of support, aged 55 years and above in 2012 were identified (ID cohort). In addition, they had to be alive at the end of that year. A one-to-one sex and age-matched control cohort from the general population (gPop cohort) was established by Statistics Sweden using the Swedish population register. People included in the ID cohort could not be included in the gPop cohort also. However, people with ID but without LSS support were not excluded from the gPop cohort.

### Outcomes

The *Swedish National Patient Register* (NPR) is also managed by the Swedish National Board of Health and Welfare. It contains information about all in- and outpatient specialist care in Sweden. However, it does not contain information about visits to primary health care. For inpatient care, registration in the NPR is made at the date of discharge, and for outpatient care it is made at the date of the visit. For each registration, one primary and up to 21 secondary diagnoses are listed, coded according to the 10th revision of the International Classification of Disease (ICD-10).

Information on psychiatric diagnoses during the study period was obtained from the NPR for 2002–2012. The mean age at the start of data collection (i.e. 1 January 2002) was 53 years (range 44–85 years). These were categorised as attention deficit/hyperactivity disorder (ADHD) and equivalents, psychotic disorders, affective disorders, anxiety disorders, personality disorders, alcohol/substance-abuse-related disorders, dementia or other psychiatric disorders (Table 1). Each person was categorised as having none or at least one diagnosis in each diagnostic category during the study period, and the date of the first record of each diagnosis for each person was noted. We did not differentiate between primary and secondary diagnoses. Thus, a secondary diagnosis of e.g. dementia would classify an individual as having at least one dementia diagnosis even if the primary diagnosis for that visit was another psychiatric diagnosis.

**Table 1.** Diagnostic category definition and number of people in a general population sample (gPop, n = 7936) and among people with intellectual disability (ID, n = 7936) with at least one diagnosis and at least one primary diagnosis, respectively, during 2002–2012

	gPop		ID	
	All	Primary	All	Primary
	*n* (%)	*n* (%)	*n* (%)	*n* (%)
Attention deficit/hyperactivity disorder (ADHD) and equivalents				
Hyperkinetic disorders (F90)	10 (0.1)	5 (0.1)	38 (0.5)	18 (0.2)
Psychotic disorders				
Schizophrenia (F20)	16 (0.2)	12 (0.2)	210 (2.6)	120 (1.5)
Schizotypal disorder (F21)	<5	<5	5 (0.1)	<5
Persistent delusional disorders (F22)	9 (0.1)	7 (0.1)	63 (0.8)	38 (0.5)
Acute and transient psychotic disorders (F23)	6 (0.1)	<5	51 (0.6)	25 (0.3)
Induced delusional disorder (F24)	<5	<5	<5	<5
Schizoaffective disorders (F25)	6 (0.1)	<5	46 (0.6)	30 (0.4)
Other nonorganic psychotic disorders (F28)	<5	<5	12 (0.2)	<5
Unspecified nonorganic psychosis (F29)	9 (0.1)	<5	128 (1.6)	60 (0.8)
Affective disorders				
Manic episode (F30)	<5	<5	32 (0.4)	19 (0.2)
Bipolar affective disorder (F31)	42 (0.5)	37 (0.5)	170 (2.1)	125 (1.6)
Depressive episode (F32)	254 (3.2)	182 (2.3)	358 (4.5)	208 (2.6)
Recurrent depressive disorder (F33)	116 (1.5)	93 (1.2)	116 (1.5)	83 (1.0)
Persistent mood [affective] disorders (F34)	23 (0.3)	14 (0.2)	21 (0.3)	11 (0.1)
Other mood [affective] disorders (F38)	5 (0.1)	<5	<5	<5
Unspecified mood [affective] disorder (F39)	17 (0.2)	10 (0.1)	35 (0.4)	17 (0.2)
Anxiety disorders				
Phobic anxiety disorders (F40)	16 (0.2)	10 (0.1)	36 (0.5)	18 (0.2)
Other anxiety disorders (F41)	196 (2.5)	131 (1.7)	289 (3.6)	219 (2.8)
Obsessive-compulsive disorder (F42)	10 (0.1)	6 (0.1)	74 (0.9)	34 (0.4)
Reaction to severe stress and adjustment disorders (F43)	143 (1.8)	116 (1.5)	81 (1.0)	66 (0.8)
Personality disorders				
Specific personality disorders (F60)	22 (0.3)	13 (0.2)	60 (0.8)	30 (0.4)
Mixed and other personality disorders (F61)	<5	<5	<5	<5
Alcohol/substance-abuse-related disorders				
Mental and behavioural disorders due to use of				
alcohol (F10)	214 (2.7)	164 (2.1)	125 (1.6)	98 (1.2)
opioids (F11)	23 (0.3)	14 (0.2)	8 (0.1)	7 (0.1)
cannabinoids (F12)	7 (0.1)	<5	6 (0.1)	<5
sedatives or hypnotics (F13)	36 (0.5)	17 (0.2)	8 (0.1)	5 (0.1)
cocaine (F14)	<5	<5	<5	<5
other stimulants, including caffeine (F15)	11 (0.1)	7 (0.1)	<5	<5
hallucinogens (F16)	1 (<0.1)	<5	<5	<5
tobacco (F17)	87 (1.1)	6 (0.1)	43 (0.5)	6 (0.1)
volatile solvents (F18)	<5	<5	<5	<5
multiple drug use and use of other psychoactive substances (F19)	33 (0.4)	21 (0.3)	19 (0.2)	11 (0.1)
Dementia				
Dementia in Alzheimer disease (F00)	24 (0.3)	15 (0.2)	51 (0.6)	26 (0.3)
Vascular dementia (F01)	18 (0.2)	11 (0.1)	22 (0.3)	<5
Dementia in other diseases classified elsewhere (F02)	<5	<5	8 (0.1)	<5
Unspecified dementia (F03)	38 (0.5)	15 (0.2)	133 (1.7)	33 (0.4)
Delirium superimposed on dementia (F05.1)	<5	<5	<5	<5
Alcoholic dementia (F10.7A)	<5	<5	<5	<5
Alzheimer disease (G30)	31 (0.4)	22 (0.3)	60 (0.8)	23 (0.3)
Other degenerative diseases of nervous system, not elsewhere classified (G31)	10 (0.1)	7 (0.1)	11 (0.1)	<5
Other psychiatric disorders				
Delirium, not induced by alcohol and other psychoactive substances (F05)	9 (0.1)	5 (0.1)	14 (0.2)	11 (0.1)
Other mental disorders due to brain damage and dysfunction and to physical disease (F06)	21 (0.3)	9 (0.1)	132 (1.7)	45 (0.6)
Personality and behavioural disorders due to brain disease, damage and dysfunction (F07)	6 (0.1)	<5	39 (0.5)	17 (0.2)
Unspecified organic or symptomatic mental disorder (F09)	<5	<5	24 (0.3)	9 (0.1)
Dissociative [conversion] disorders (F44)	<5	<5	27 (0.3)	18 (0.2)
Somatoform disorders (F45)	48 (0.6)	34 (0.4)	31 (0.4)	22 (0.3)
Other neurotic disorders (F48)	5 (0.1)	<5	11 (0.1)	6 (0.1)
Eating disorders (F50)	<5	<5	7 (0.1)	<5
Nonorganic sleep disorders (F51)	16 (0.2)	4 (0.1)	10 (0.1)	<5 (<0.1)
Sexual dysfunction, not caused by organic disorder or disease (F52)	<5	<5	<5	<5
Mental and behavioural disorders associated with the puerperium, not elsewhere classified (F53)	<5	<5	<5	<5
Habit and impulse disorders (F63)	<5	<5	10 (0.1)	<5
Disorders of sexual preference (F65)	<5	<5	5 (0.1)	<5
Other disorders of adult personality and behaviour (F68)	<5	<5	<5	<5
Unspecified disorder of adult personality and behaviour (F69)	<5	<5	10 (0.1)	<5
Specific developmental disorders of speech and language (F80)	<5	<5	68 (0.9)	<5
Specific developmental disorders of scholastic skills (F81)	<5	<5	11 (0.1)	<5
Mixed specific developmental disorders (F83)	<5	<5	<5	<5
Unspecified disorder of psychological development (F89)	<5	<5	395 (5.0)	96 (1.2)
Conduct disorders (F91)	<5	<5	13 (0.2)	6 (0.1)
Mixed disorders of conduct and emotions (F92)	<5	<5	<5	<5
Emotional disorders with onset specific to childhood (F93)	<5	<5	<5	<5
Disorders of social functioning with onset specific to childhood and adolescence (F94)	<5	<5	<5	<5
Tic disorders (F95)	<5	<5	16 (0.2)	<5
Other behavioural and emotional disorders with onset usually occurring in childhood and adolescence (F98)	<5	<5	23 (0.3)	<5
Mental disorder, not otherwise specified (F99)	<5	<5	31 (0.4)	<5
Auditory hallucinations (R44.0)	<5	<5	12 (0.2)	<5

Numbers are not given for cells where there are less than five observations.

### Statistics

To compare the number of people with at least one of each respective category of psychiatric diagnoses in the ID cohort to the corresponding number in the gPop cohort, we estimated odds ratios (ORs) with 95% confidence intervals (CIs) using logistic regression. In order to illustrate possible age-effects, we performed age stratified analyses, using the 5-year age categories. Moreover, we investigated age effects within the ID cohort by comparing each age group to the youngest one. Statistical interaction was evaluated by introducing an interaction term (e.g. cohort*age group) to the logistic regression model, and trends were assessed by treating the category variable as a continuous factor.

In order to evaluate using the LSS register as a proxy for ID, we performed sensitivity analyses on sub-cohorts of people with known diagnosis of either ID or ASD. Through diagnoses available from the NPR, we were able to identify 1145 men and 1002 women who had at least one diagnosis of ID (F7 in ICD-10) during 2002–2012. Moreover, we identified 242 men and 156 women who had at least one diagnosis of ASD (F84, excluding F84.1 and F84.5, in ICD-10). The overlap was 209 individuals. Analyses were made comparing psychiatric diagnoses among those with ASD only to those with ID only or ASD in combination with ID. Also, as people with ID may be difficult to diagnose with respect to psychiatric disorders, we performed sensitivity analyses including only diagnoses recorded at psychiatric clinics, i.e. by psychiatric specialists.

Analyses were only performed if each of the two compared group comprised at least five observations. A two-tailed *p*-value of 0.05 was considered statistically significant. All analyses were performed in IBM SPSS Statistics 23.

## Results

Each cohort comprised 7936 people, whereof 45% were women. The percentage of women increased over the age categories, with 43% among those aged 55–59 years (total *n* = 2559 in each cohort), 44% among those aged 60–64 years (*n* = 2097), 46% among those aged 65–69 years (*n* = 1636), 48% among those aged 70–74 years (*n* = 839) and 53% among those aged 75+ years (*n* = 805).

**Fig. 1.**
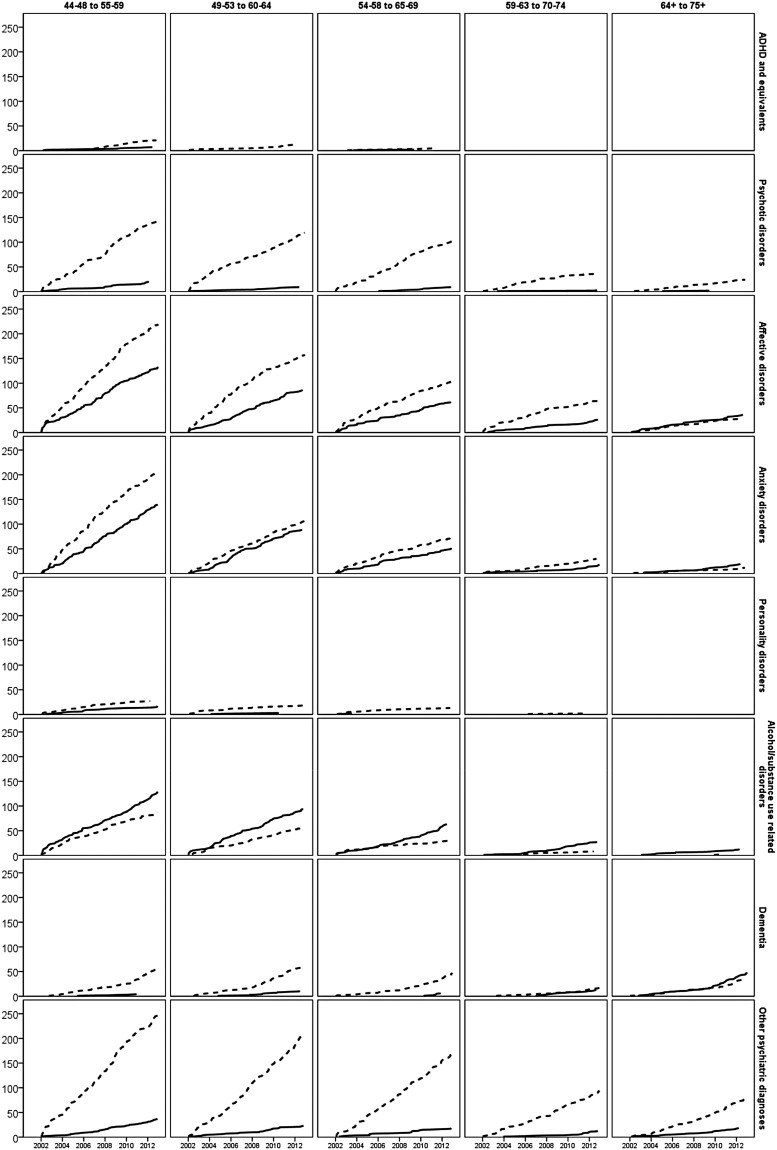
Cumulative number of people with diagnoses within different diagnostic categories during 2002–2012 in a cohort of 7936 people with ID (broken lines) and a same-sized sample from the general population, one-to-one matched by age and sex (gPop, solid lines).

Figure 1 describes the cumulative number of people with a recorded diagnosis during the study period, by cohort, age group and diagnostic category. The rightmost end of each line corresponds to the number of people for whom each diagnosis was recorded at least once during the study period, which is also given in Table 2. Increased ORs for the ID cohort were found for all diagnostic categories except alcohol/substance-abuse-related disorders. The ORs that indicated increased odds for the ID cohort ranged from 1.36 (anxiety disorders) to 10.4 (psychotic disorders). Sensitivity analyses including only diagnoses recorded in psychiatric care increased the OR for dementia by 60% (from 2.71 to 4.34), for ‘other psychiatric diagnosis’ by 29% (from 8.02 to 10.32), for any psychiatric diagnosis by 24% (from 1.84 to 2.28), and for anxiety disorders by 16% (from 1.36 to 1.58). For all other diagnostic categories, the results remained similar (less than 15% change; ORs not shown).

**Table 2.** Number of people with at least one diagnosis in different psychiatric diagnostic categories during 2002–2012, among people with intellectual disability (ID) and a one-to-one age- and sex-matched sample from the general population (gPop)

	ADHD^a^	Psychotic disorders	Affective disorders	Anxiety disorders	Personality disorders	Alcohol/substance-abuse-rel. disorders	Dementia	Other psychiatric disorders	Any psychiatric disorder
All (*n* = 7936 in each cohort)									
gPop, *n* (%)	1<5	43 (1)	342 (4)	317 (4)	23 (0)	325 (4)	81 (1)	109 (1)	817 (10)
ID, *n* (%)	38 (0)	425 (5)	576 (7)	425 (5)	61 (1)	186 (2)	216 (3)	797 (10)	1382 (17)
ID *v.* gPop	**3.81 (1.90–7.66)**	**10.4 (7.58–14.2)**	**1.74 (1.51–1.99)**	**1.36 (1.17–1.58)**	**2.66 (1.65–4.31)**	**0.56 (0.47–0.67)**	**2.71 (2.10–3.51)**^b^	**8.02 (6.55–9.82)**	**1.84 (1.68–2.02)**
Specialist^c^	**5.35 (2.24–12.8)**	**11.9 (8.05–17.7)**	**1.66 (1.43–1.93)**	**1.33 (1.14–1.57)**	**2.25 (1.34–3.76)**	**0.60 (0.48–0.75)**	**2.36 (1.72–3.24)**	**5.28 (4.15–6.72)**	**1.84 (1.66–2.04)**
44–48 in 2002 to 55–59 in 2012 (*n* = 2559 in each cohort)									
gPop, *n* (%)	7 (0)	20 (1)	133 (5)	140 (5)	17 (1)	129 (5)	<5	37 (1)	291 (11)
ID, *n* (%)	21 (1)	143 (6)	219 (9)	205 (8)	27 (1)	87 (3)	56 (2)	247 (10)	526 (21)
ID *v.* gPop	**3.02 (1.28–7.11)**	**7.51 (4.69–12.0)**	**1.71 (1.37–2.13)**	**1.50 (1.20–1.88)**	1.59 (0.87–2.93)	**0.66 (0.50–0.88)**	NC	**7.28 (5.13–10.3)**	**2.02 (1.73–2.36)**
49–53 in 2002 to 60–64 in 2012 (*n* = 2097 in each cohort)									
gPop, *n* (%)	<5	9 (0)	86 (4)	89 (4)	<5	94 (4)	1<5	23 (1)	213 (10)
ID, *n* (%)	12 (1)	119 (6)	157 (7)	106 (5)	18 (1)	56 (3)	60 (3)	209 (10)	374 (18)
ID *v.* gPop	NC	**14.0 (7.07–27.6)**	**1.89 (1.44–2.48)**	1.20 (0.90–1.60)	NC	**0.58 (0.42–0.82)**	**6.15 (3.14–12.0)**	**9.98 (6.46–15.4)**	**1.92 (1.60–2.30)**
54–58 in 2002 to 65–69 in 2012 (*n* = 1636 in each cohort)									
gPop, *n* (%)	<5	9 (1)	61 (4)	51 (3)	<5	63 (4)	6 (0)	18 (1)	141 (9)
ID, *n* (%)	5 (0)	102 (6)	105 (6)	72 (4)	13 (1)	32 (2)	47 (3)	170 (10)	272 (17)
ID *v.* gPop	2.50 (0.49–12.9)	**12.0 (6.06–23.8)**	**1.77 (1.28–2.45)**	1.43 (0.99–2.06)	NC	**0.50 (0.32–0.77)**	**8.04 (3.43–18.8)**	**10.4 (6.38–17.0)**	**2.11 (1.70–2.62)**
59–63 in 2002 to 70–74 in 2012 (*n* = 839 in each cohort)									
gPop, *n* (%)	<5	<5	26 (3)	18 (2)	<5	27 (3)	13 (2)	12 (1)	77 (9)
ID, *n* (%)	<5	37 (4)	67 (8)	30 (4)	<5	8 (1)	17 (2)	94 (11)	123 (15)
ID *v.* gPop	NC	NC	**2.71 (1.71–4.31)**	1.69 (0.94–3.06)	NC	**0.29 (0.13–0.64)**	1.31 (0.63–2.72)	**8.70 (4.73–16.0)**	**1.70 (1.26–2.30)**
64+ in 2002 to 75+ in 2012 (*n* = 805 in each cohort)									
gPop, *n* (%)	<5	<5	36 (4)	19 (2)	<5	12 (1)	48 (6)	19 (2)	95 (12)
ID, *n* (%)	<5	24 (3)	28 (3)	12 (1)	<5	<5	36 (4)	77 (10)	87 (11)
ID *v.* gPop	NC	NC	0.77 (0.47–1.27)	0.63 (0.30–1.30)	NC	NC	0.74 (0.47–1.15)	**4.38 (2.62–7.30)**	0.91 (0.67–1.23)
*p*_interaction_	0.696	0.277	0.318	0.198	0.107	**0.026**	**<0.001**	0.310	**<0.001**

NC, not calculated as at least one cell contains less than five observations.

Comparisons of ID *v.* gPop are done using logistic regression, thus estimating OR with 95% confidence intervals. p values relate to the possible interaction between age group and ID. Statistically significant results are marked in bold.

a Attention deficit/hyperactive disorder.

b Originally presented in Axmon *et al.* (2016).

c Subgroup analyses including only diagnoses recorded in specialist care.

When stratifying by age group, the results were consistent with those found for the whole cohorts for ADHD as well as psychotic, affective, anxiety, personality and ‘other’ psychiatric disorders. However, for alcohol/substance-abuse-related disorders, the OR for the ID cohort *v.* the gPop cohort decreased, such that it moved away from the null, with increasing age. Moreover, for dementia and any psychiatric disorder, the OR for the ID cohort *v.* the gPop cohort decreased towards the null with age.

Within the ID cohort, the oldest age group had lower odds than the youngest age group of psychotic, affective, and anxiety disorders, as well as of dementia and any psychiatric diagnosis (Table 3). No such effect was found for ‘other’ psychiatric diagnoses. We could not perform the corresponding analyses for ADHD, personality disorders, or alcohol/substance-abuse-related disorders as the number with recorded diagnoses in the oldest age group was too low. However, the results from the two middle age groups suggested that the odds of diagnoses of ADHD or alcohol/substance-abuse-related disorders also increased with age. In the sensitivity analyses, those with ASD only had lower odds of receiving psychiatric diagnoses than those with ID only or those with ID in combination with ASD, with exception of diagnoses of ADHD (Table 4). However, statistical significance was achieved only for affective disorders, ‘other psychiatric diagnoses’ (only in comparison with ID and ASD in combination), and ‘any’ psychiatric diagnosis.

**Table 3.** Number of people identified through the LSS register with at least one diagnosis in different psychiatric diagnostic categories in inpatient or specialist outpatient care during 2002–2012, grouped according to age in 2002

	44–48 years (*n* = 2559)	49–53 years (*n* = 2097)		54–58 years (*n* = 1636)		59–63 years (*n* = 839)		64+ years (*n* = 805)		
	*n* (%)	*n* (%)	OR (95% CI)	*n* (%)	OR (95% CI)	*n* (%)	OR (95% CI)	*n* (%)	OR (95% CI)	*p*_trend_
Attention deficit/hyperactivity disorder (ADHD)	21 (1)	12 (1)	0.70 (0.34–1.42)	5 (0)	**0.37** (**0.14–0.99)**	<5	NC	<5	NC	**<0**.**001**
Psychotic disorders	143 (6)	119 (6)	1.02 (0.79–1.31)	102 (6)	1.12 (0.87–1.46)	37 (4)	0.79 (0.54–1.13)	24 (3)	**0.52 (0.33–0.81)**	**0.014**
Affective disorders	219 (9)	157 (7)	0.87 (0.70–1.07)	105 (6)	**0.73 (0.58–0.93)**	67 (8)	0.93 (0.70–1.23)	28 (3)	**0.39 (0.26–0.58)**	**<0.001**
Anxiety disorders	205 (8)	106 (5)	**0.61 (0.48–0.78)**	72 (4)	**0.53 (0.40–0.70)**	30 (4)	**0.43 (0.29–0.63)**	12 (1)	**0.17 (0.10–0.31)**	**<0.001**
Personality disorders	27 (1)	18 (1)	0.81 (0.45–1.48)	13 (1)	0.75 (0.39–1.46)	<5	NC	<5	NC	**0.002**
Alcohol/substance-abuse-related disorders	87 (3)	56 (3)	0.78 (0.55–1.10)	32 (2)	**0.57 (0.38–0.85)**	8 (1)	**0.27 (0.13–0.57)**	<5	NC	**<0.001**
Dementia	56 (2)	60 (3)	1.32 (0.91–1.90)	47 (3)	1.32 (0.89–1.96)	17 (2)	0.92 (0.53–1.60)	36 (4)	**2.09 (1.37–3.21)**	**0.014**
Other psychiatric diagnoses	247 (10)	209 (10)	1.04 (0.85–1.26)	170 (10)	1.09 (0.88–1.33)	94 (11)	1.18 (0.92–1.52)	77 (10)	0.99 (0.76–1.30)	0.50
Any psychiatric diagnosis	526 (21)	374 (18)	**0.84 (0.72–0.97)**	272 (17)	**0.77 (0.66–0.91)**	123 (15)	**0.66 (0.54–0.82)**	87 (11)	**0.47 (0.37–0.60)**	**<0.001**

NC, not calculated as at least one cell contains less than five observations.

Comparisons of age groups are done using logistic regression with the youngest age group as referent group, thus estimating odds ratios (ORs) with 95% confidence intervals (CIs). Statistically significant results are marked in bold.

**Table 4.** Number of people identified through the LSS register with at least one diagnosis in different psychiatric diagnostic categories in inpatient or specialist outpatient care during 2002–2012, grouped according to diagnosis of intellectual disability (ID, F7 in ICD-10) and autism spectrum disorder (ASD, F84, excluding F84.1 and F84.5, in ICD-10)

	ASD diagnosis only (*n* = 189)	ID diagnosis only (*n* = 1938)		Both ID and ASD diagnoses (*n* = 209)	
	*n* (%)	*n* (%)	OR (95% CI)	*n* (%)	OR (95% CI)
Attention deficit/hyperactivity disorder (ADHD)	3 (2)	12 (1)	0.39 (0.11–1.38)	3 (1)	0.90 (0.18–4.53)
Psychotic disorders	19 (10)	293 (15)	1.59 (0.98–2.60)	24 (11)	1.16 (0.61–2.19)
Affective disorders	20 (11)	332 (17)	**1.75 (1.08–2.82)**	41 (20)	**2.06 (1.16–3.67)**
Anxiety disorders	14 (7)	213 (11)	1.54 (0.88–2.71)	28 (13)	1.93 (0.99–3.80)
Personality disorders	2 (1)	27 (1)	1.32 (0.31–5.60)	2 (1)	0.90 (0.13–6.48)
Alcohol/substance-abuse-related disorders	5 (3)	62 (3)	1.22 (0.48–3.06)	3 (1)	0.54 (0.13–2.27)
Dementia	4 (2)	84 (4)	2.10 (0.76–5.78)	9 (4)	2.08 (0.63–6.87)
Other psychiatric diagnoses	30 (16)	373 (19)	1.26 (0.84–1.90)	62 (30)	**2.24 (1.37–3.65)**
Any psychiatric diagnosis	45 (24)	727 (38)	**1.92 (1.36–2.72)**	80 (38)	**1.98 (1.28–3.07)**

Statistically significant results are marked in bold.

## Discussion

Older people with ID had higher odds than their counterparts in the general population to have at least one psychiatric diagnosis recorded in inpatient or outpatient specialist care. The largest discrepancy between the two cohorts was for psychotic disorders, of which people with ID had more than ten times the odds of having a diagnosis recorded during the 11-year study period. The only diagnostic category for which a higher odds was found in the general population was alcohol/substance-abuse-related disorders.

The ID cohort is an administratively identified group of people with ID in Sweden, using the LSS-register. The people in this group were 55 years and older in 2012, and thereby belong to the group of people with ID who received services before the LSS Act (SFS 1993:387, 1993) was passed in 1993. Prior to this, people who had a diagnosis of ID were more or less automatically registered for service based on their diagnosis, and not as in the current act, after applying for support. Therefore, the cohort can be expected to fairly well cover the group of ageing people with ID in Sweden. Nevertheless, the register does not contain information on the diagnosis that was the basis for the provision of services (i.e. ID or ASD). Thereby we cannot distinguish those who have ID only or ID in combination with ASD from those who have ASD only. Sensitivity analyses showed that those with ASD only had lower odds than those with ID only or ID in combination with ASD to have different psychiatric diagnoses recorded in the NPR. Thus, the higher the fraction of people with ASD only in the ID cohort, the more likely we would be to underestimate the effect of having ID.

It is important not to confuse psychiatric diagnoses with psychiatric disorders. Whereas a disorder relates to the actual state of health of a person, a diagnosis is merely a proxy thereof. If it is more likely that a disorder will go undiagnosed than it is that a diagnosis is not linked to a disorder in a person, the number of diagnoses will tend to underestimate the true numbers, especially in non-chronic disorders. The opposite is true if diagnoses are given without an underlying disorder, e.g. if a behaviour resulting from environmental stressors is misinterpreted as symptoms of a psychiatric disorder.

The numbers provided in this study are prevalences for the study period. For chronic disorders, these prevalences may be used as estimates of point prevalence at the study end, and may therefore be compared with point prevalences presented in other studies. However, for non-chronic disorders, such comparisons are not meaningful.

Diagnosing psychiatric disorders in people with ID may be difficult, as the patient must not only recognise the symptoms but also be able to communicate them, which is not always the case, especially among those with severe or profound ID. Diagnosing is further complicated by diagnostic overshadowing (Reiss & Szyszko, 1983), i.e. when professionals attribute the symptoms of the psychiatric disorder to the ID, and masking, i.e. when the clinical characteristics of the psychiatric disorder are considered secondary to the ID. As a consequence of these diagnostic challenges, a large part of psychiatric morbidity in people with ID is hidden (Salvador-Carulla *et al.* 2000), which may lead to an undercount in health care among people with ID. For some psychiatric disorders, assessment tools are available, at least for people with mild or moderate ID (Mindham & Espie, 2003; Deb *et al.* 2007; Antonacci & Attiah, 2008; Perez-Achiaga *et al.* 2009; Havercamp & Scott, 2015). For others, researchers have called for further development and evaluation of diagnostic systems for people with ID (Alexander & Cooray, 2003; Moreland *et al.* 2008; Hermans & Evenhuis, 2010). This is important to be aware of when comparing the occurrence of psychiatric diagnoses among people with ID to groups with more communicative skills, such as the general population.

There are different systems to classify psychiatric disorders among people with ID. Diagnoses in the Swedish NPR, on which the present study is based, are classified according to the ICD-10. However, diagnoses may also be made according to e.g. the Diagnostic and Statistical Manual of Mental Disorders (DSM) or the Diagnostic Criteria for psychiatric disorders for use with adults with Learning Disabilities/mental retardation (DC-LD). These are not identical with respect to diagnostic criteria, and so diagnoses may differ between them. Differences between diagnostic criteria have been found both in the general population (Erkinjuntti *et al.* 1997; Cheniaux *et al.* 2009; Nilsson *et al.* 2012) and among people with ID (Cooper *et al.* 2007*b*; Mantry *et al.* 2008; Melville *et al.* 2008; Strydom *et al.* 2008). If a diagnostic system is more or less likely to diagnose a person with ID than one from the general population, the potential risk of having the diagnosis associated with having ID would not be correctly estimated.

The training and experience of the professional making the diagnosis may also contribute to differences in diagnoses between groups. In order to investigate whether this may be an issue in the present study, we performed sensitivity analyses including only diagnoses recorded in psychiatric care. In the sensitivity analyses, ORs associated with ID increased for dementia, anxiety disorders, ‘other’ psychiatric disorders and any psychiatric disorder. If psychiatric diagnoses made in psychiatric care are more correct than diagnoses recorded in somatic care, this implies that these disorders may go undiagnosed if the person with ID is treated only in somatic clinics.

Over the 11-year study period, people with ID had three times higher odds than those in the general population to have *at least one psychiatric diagnosis* recorded in inpatient or outpatient specialist care. Previous studies have investigated prevalence of psychiatric diagnoses among adults up to 64 years of age and with mild or moderate ID, using diagnoses according to the ICD-10 (Deb *et al.* 2001; Schutzwohl *et al.* 2016). When comparing these prevalences to those found in the general population, neither of them found an increased prevalence associated with ID. In studies including people older than 65 years and without restrictions regarding severity of ID, different patterns have been found. Cooper *et al.* (2007*b*) found a higher prevalence among people with ID than what was found in the general population in the UK when diagnosing according to the DC-LD, but not when using ICD-10 or DSM-IV. Nettelbladt *et al.* (2009) found a 34% increased (based on cumulative incidence) for psychiatric diagnoses according to DSM-IV among people with ID compared with those without. Also, Cooper *et al.* (2015) found that the prevalence of psychiatric diagnoses according to the READ code were twice as high among people with ID as among those without ID. The effect found in the present study is slightly larger than that found in these studies. This discrepancy may be explained by the differences in age distributions in the three studies, as old age, at least up until 75 years, has been found to be associated with higher rates of psychiatric morbidity among people with ID (Cooper, 1997*b*; Cooper *et al.* 2015). Differences in diagnostic criteria are also likely to account for some of the discrepancy, as is differences in the outcome measures (e.g. prevalence *v.* incidence).

The percentage of people with ID who had at least one psychiatric diagnosis during the study period decreased with age. Even though life expectancy is increasing among people with ID, it is still shorter than in the general population. Thus, the decrease in diagnoses among the oldest in the ID cohort may, at least partly, be due to a survival bias, i.e. that the oldest age group is healthier simply because those who had severe disorders would not have been included in the present study (as being alive was an inclusion criteria).

The largest OR for people with ID compared with those in the general population in the present study was found for diagnoses of *psychotic disorders*. This is well in line with previous studies investigating people with and without ID, where a considerable increase in overall psychotic disorders and schizophrenia specifically has been found for people with ID, regardless of which diagnostic system was used (Deb *et al.* 2001; Cooper *et al.* 2007*a*, 2015; Gentile *et al.* 2014; Howlett *et al.* 2015; Carey *et al.* 2016).

The 74% increased odds for diagnoses of *affective disorders* among people with ID in the present study is similar to what has been found in other community-based populations (Deb *et al.* 2001) and administratively defined cohorts (Howlett *et al.* 2015). However, Carey *et al.* (2016) found an almost sevenfold increased prevalence of affective disorders among people with ID in comparison with the general population when studying data from primary care. The different results may in part be explained by the use of different diagnostic systems or different outcome measures. However, it may also by that although people with ID may have increased risk of affective disorders, they are not likely to receive specialist care for this type of psychiatric diagnosis.

We found diagnoses of *anxiety disorders* to be more common among people with ID than in the general population, which is in line with some (Gentile *et al.* 2014; Carey *et al.* 2016), but not all (Deb *et al.* 2001; Hermans *et al.* 2013; Howlett *et al.* 2015), previous studies. One explanation to the different results found may be the use of different diagnoses systems or outcome measures. However, there are also other factors that may account for at least part of the discrepancy. The rate of anxiety disorders increases with age among people with ID (Cooper, 1997*a*; Hermans *et al.* 2013), but decreases with age in the general population (Ramsawh *et al.* 2009; Blay & Marinho, 2012). Thus, the ratio of anxiety disorders among people with ID and the general population should increase with age. This could explain the discrepancy in results between the present study and that by Deb *et al.* (2001) and Howlett *et al.* (2015), as the people in these studies were younger than those in the present. The study population in (Hermans *et al.* (2013) had, however, a similar age distribution as our study group. A drawback of that study was the use of a standardised interview not adapted to people with ID, which may underestimate the true prevalence of anxiety disorders in the group of people with ID.

Although several studies have investigated *personality disorders* among criminal offenders with ID, not much research has been published on this dual diagnosis in a more general ID population. The limited evidence available suggests that people with ID are at a greater risk for diagnoses of personality disorders (Pridding & Procter, 2008; Howlett *et al.* 2015). The increase in diagnoses of personality disorders in the present study is thereby in agreement with and adds important information to the knowledge base regarding personality disorders among people with ID.

People with ID seem to be more sensitive than the general population to developing a *substance-abuse-related disorder* (McGillicuddy & Blane, 1999; Chapman & Wu, 2012; van Duijvenbode *et al.* 2015). In the present study, only two percent of those with ID had a diagnosis of alcohol/substance-abuse-related disorders. This is at the low end of previously published results (Cooper *et al.* 2015; van Duijvenbode *et al.* 2015). As substance abuse is not necessarily a chronic state, the 11-year prevalence would be expected to be higher than point prevalences. Differences in the definition and selection of ID-group, study design, age groups studied, living conditions, severity of ID and definition of substance use may contribute to the variation in numbers. We found an almost threefold increase in odds of *dementia* diagnosis associated with having ID. Cooper *et al.* (2015) and Carey *et al.* (2016) both used primary care data to identify people with ID as well as diagnosis of dementia according to READ code. In both studies, about a fourth of the group of people with ID were aged 55 years, or older, and both studies found a large increase in risk of dementia among those with ID compared with the general population. However, Gentile *et al.* (2014) who let a psychiatrist diagnose all participants, found lower prevalence of dementia among people with ID compared with the population prevalence when using data from outpatient clinics. As the authors do not provide the age of the participants, age differences may be a possible explanation for the discrepancy with other studies. Another explanation may be that people with ID and dementia are more often treated in primary care, or that different diagnostic systems identify dementia differently in people with ID.

In the present study, *ADHD* was the least frequent psychiatric diagnosis among people with ID, with less than one percent of the cohort getting a diagnosis during the study period. As people in this age group are unlikely to ‘lose’ their ADHD diagnosis, this number may be used as a prevalence estimate. Compared with other studies, it is a low one (Fox & Wade, 1998; La Malfa *et al.* 2008). This may partly be explained by differences in age distributions, and that primary care is not included in the present study. However, it cannot be ruled out that ADHD is underdiagnosed among older people with ID in Sweden.

The co-existence of ID and psychiatric disorders does not only have a negative impact on the individual, but also places a burden on the health care system and family members. Therefore, further research into the understanding of diagnosis and treatment of such disorders in this vulnerable group of people is vital.

## Availability of Data and Materials

The data in the present study contain sensitive information on a very vulnerable group, i.e. people with ID. Even though the data are anonymised, it contains enough details to enable identification of single individuals. Therefore, in order to approve the study, the Regional Ethical Review Board in Lund made considerable restrictions regarding access to the data. This means we will not be able to provide other researchers with our data. However, as our database is compiled by register data only, other researchers may contact Statistics Sweden and the Swedish National Board of Health and Welfare to get access to the different registers included, and thereby recreate the database.
